# PDGFRα-positive mesenchymal stem/stromal cells contribute to autonomous vascular formation through in-body tissue architecture

**DOI:** 10.1371/journal.pone.0347197

**Published:** 2026-04-16

**Authors:** Satoru Morikawa, Masaki Yoda, Takehito Ouchi, Takazumi Yasui, Kazumasa Fukuda, Michiru Sugimoto, Rintaro Harada, Tatsuaki Matsumoto, Yo Mabuchi, Osahiko Tsuji, Narihito Nagoshi, Yoshitaka Kase, Hideyuki Okano, Yuko Kitagawa, Masaya Nakamura, Taneaki Nakagawa, Yasuhide Nakayama

**Affiliations:** 1 Department of Dentistry and Oral Surgery, Keio University School of Medicine, Shinjuku-ku, Tokyo, Japan; 2 Department of Orthopaedic Surgery, Keio University School of Medicine, Shinjuku-ku, Tokyo, Japan; 3 Department of Physiology, Tokyo Dental College, Chiyoda-ku, Tokyo, Japan; 4 Department of Surgery, Keio University School of Medicine, Shinjuku-ku, Tokyo, Japan; 5 School of Medicine, Fujita Health University, Aichi, Japan; 6 Division of CNS Regeneration and Drug Discovery, International Center for Brain Science (ICBS), Fujita Health University, Toyoake-Shi, Aichi, Japan; 7 Keio University Regenerative Medicine Research Center, Kawasaki-ku, Kawasaki, Japan; 8 Department of Geriatric Medicine, Graduate School of Medicine, The University of Tokyo, Bunkyo-ku, Tokyo, Japan; 9 Osaka Laboratory, Biotube Co., Ltd., Osaka, Japan; University of Minnesota Medical School, UNITED STATES OF AMERICA

## Abstract

Driven by endogenous platelet-derived growth factor receptor α (PDGFRα)-positive cells, in-body tissue architecture (iBTA) enables the autonomous formation of vascularized tissues within subcutaneously implanted molds, overcoming the limitations of traditional cell therapies. The cellular mechanisms were investigated in PDGFRα reporter mice. Recruited PDGFRα-lineage cells were closely associated with Pecam-1-positive vessels and often adopted perivascular positions. Flow cytometry revealed that these cells expressed the mesenchymal stem/stromal cell (MSC) markers (CD73, CD90, and CD105). Additionally, the cultured isolates maintained MSC morphology and demonstrated osteogenic, chondrogenic, and adipogenic differentiation potential in vitro. The approach was successfully scaled to a porcine model, which exhibited organized tissue maturation, including vascular structures and collagen deposition, within 2 weeks. iBTA eliminates the need for cell isolation and immunosuppression by leveraging resident PDGFRα-positive MSCs for in situ tissue generation, providing a direct pathway for autologous vascular tissue engineering applications.

## Introduction

Mesenchymal stem/stromal cells (MSCs) are of particular interest for regenerative medicine applications due to their multipotent differentiation and immunomodulatory capabilities [1,2]. Their applications range from wound healing [3,4] to bone repair [5–7]. Although the in vitro expansion and characterization of MSCs based on methods such as plastic adherence and surface marker expression (CD73+, CD90+, and CD105+) [8] have enabled progress, the mechanism by which MSCs function within their native tissue context in vivo has not been fully elucidated. Understanding the identity, recruitment [9–11], and behavior of endogenous MSCs is crucial to optimizing regenerative therapies and overcoming challenges associated with ex vivo manipulation, such as altered cell properties and immunogenicity [12]. The specific cell populations that contribute to tissue regeneration in response to biomaterials or injury in situ remain unknown [13].

In-body tissue architecture (iBTA) technology offers a unique platform for studying in vivo processes. By implanting specially designed molds, iBTA guides endogenous cells to form organized tissues in situ without prior cell isolation or culture, such as vascular [14] and tracheal structures [15]. Autologous tissues generated via iBTA (“biosheets”) have demonstrated promising clinical results in challenging applications, such as diabetic foot ulcer treatment [16], highlighting the potent regenerative capacity elicited by this approach. Previous analyses have revealed that the resulting tissue is immature and contains primitive stem cells [17,18]. However, the fundamental question of which specific endogenous cells orchestrate this in vivo tissue assembly and vascularization remains unanswered. In this context, iBTA molds can be conceptualized as a scaffold component within the classical tissue engineering paradigm of scaffolds, growth factors, and stem cells. Rather than aiming to produce a finished vascular graft, the present study leverages the iBTA system as an in vivo platform to elucidate the cellular mechanisms underlying autonomous tissue vascularization.

To address this gap, we focused on identifying key cellular players using prospective in vivo markers to circumvent the limitations of in vitro-based definitions. Platelet-derived growth factor receptor α (PDGFRα) has been established as a reliable marker for directly identifying and isolating multipotent MSCs from native murine bone marrow (PDGFRα^+^/Sca-1^+^/CD45^−^/TER119^−^) [19]. PDGFRα-positive cells occupy perivascular niches and play roles in tissue organization and vascular development [20–25]. Based on these characteristics, we hypothesized that they contribute to iBTA-mediated vasculogenesis. Despite the availability of PDGFRα lineage tracing tools [20,26], their specific use in tracking MSC behavior within the iBTA model remains unexplored.

Therefore, this study aimed to investigate the role of PDGFRα-positive cells in autonomously generating vascularized tissue within the iBTA system using PDGFRα reporter mice and to systematically track the recruitment and spatial organization of endogenous cells in relation to the development of vascular networks after mold implantation. Furthermore, this study aimed to assess the feasibility of this process in a large animal model using domestic pigs. The findings provide valuable insights into the cellular basis of iBTA-driven regeneration and highlight its potential as an autologous, cell culture-free approach for vascular tissue engineering.

## Results

### Subcutaneous implantation of iBTA molds resulted in internal tissue formation

**Fig 1:**
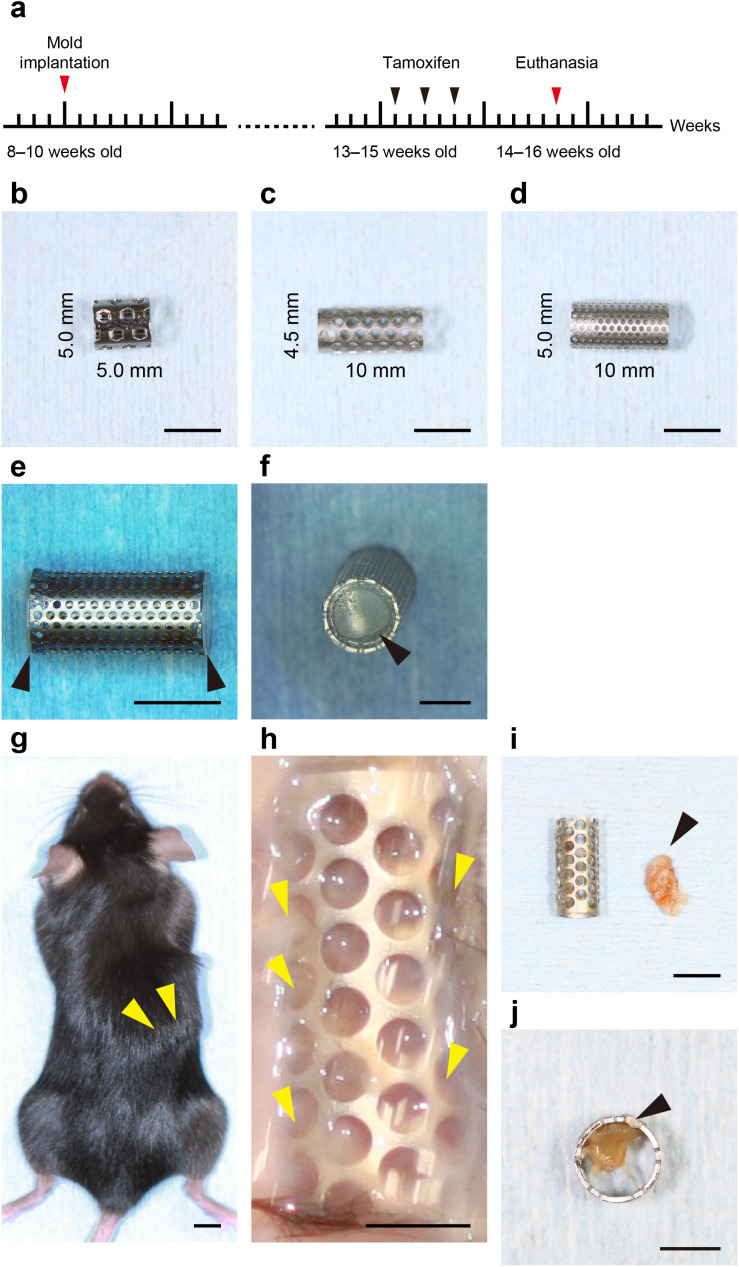
Preparation and implantation of iBTA molds into murine subcutaneous tissues. (a) Experimental timeline for mold implantation, tamoxifen administration (reporter mice), and tissue harvest. (b–d) Images of different mold configurations used. The mold dimensions were (b) 5.0 × 5.0 mm (standard type, square perforations), (c) 4.5 × 10 mm (extended type, diamond perforations), and (d) 5.0 × 10 mm (extended type, perforation pattern shown). Scale bar = 5.0 mm. (e, f) The implantable mold consists of a porous stainless steel cylinder with silicone end caps (black arrowheads) designed to permit cell entry only through the side micropores. Scale bar = 5.0 mm. (g) Appearance of the implanted mold beneath the dorsal skin before dissection (yellow arrowheads). Scale bar = 5.0 mm. (h) Harvested mold encapsulated in thin membranous tissue (yellow arrowhead). Scale bar = 2.5 mm. (i) Cross-section of the harvested mold showing successful tissue formation within the internal space (black arrowhead). Scale bar = 5.0 mm. (j) Internal tissue formation is hindered due to pore obstruction caused by excessive growth of the outer encapsulating membrane (black arrowheads). Scale bar = 5.0 mm.

Cylindrical iBTA molds were harvested for analysis after subcutaneous implantation in mice for 6 weeks (Fig 1a). Three different mold configurations were tested: a standard type (5.0 × 5.0 mm, square perforations; Fig 1b) and two extended types (4.5 × 10 mm, diamond perforations, Fig 1c; 5.0 × 10 mm, perforations detailed in Fig 1d). These porous stainless steel molds, sealed with silicone end caps (Fig 1e and f), allowed internal tissue formation via cellular infiltration through the wall micropores. The implants were well tolerated and remained in place without adverse events noted on external observation (Fig 1g). At harvest, a thin membranous capsule surrounded the molds (Fig 1h). Cross-sectional analysis revealed the presence of newly formed tissue within the internal spaces of most molds (Fig 1i). However, the robust formation of the outer encapsulating membrane physically blocked the micropores and prevented sufficient cell entry, thereby hindering tissue formation within the mold cavity (Fig 1j).

### PDGFRα-positive cells were associated with the development of vascular networks

**Fig 2:**
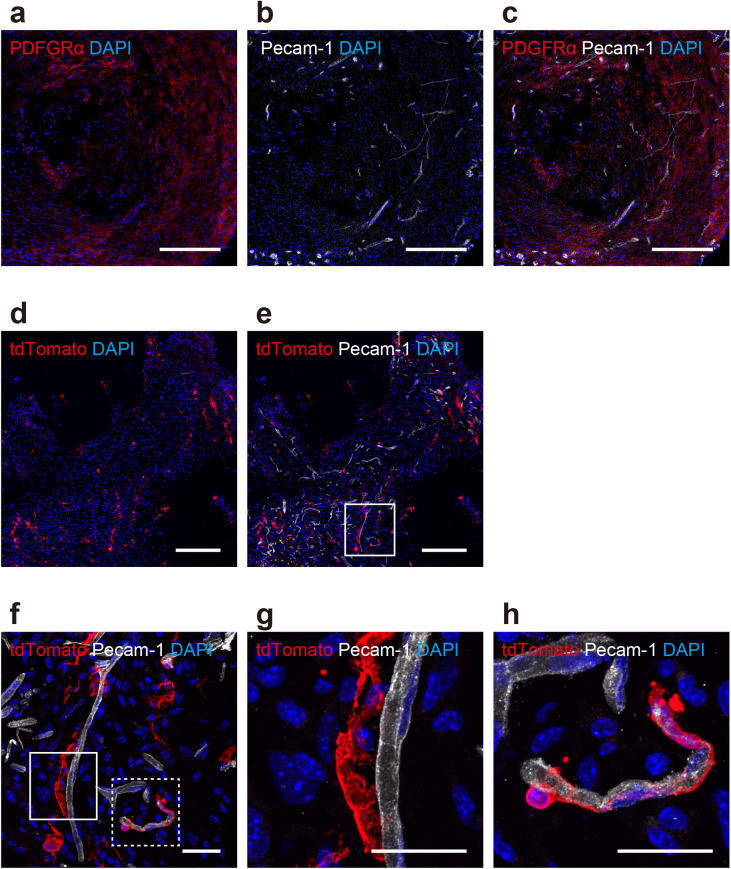
PDGFRα-positive cells associated with vascular networks in mold-derived tissues. Immunofluorescence staining of tissues harvested 6 weeks after implantation (representative of n = 3 wild-type mice for panels a–c, and n = 3 PDGFRα reporter mice for panels d–h). Nuclei were counterstained with DAPI (blue). (a–c) Analysis of wild-type mice. (a) PDGFRα-positive cells (red). (b) Pecam-1-positive endothelial vascular structures (white). (c) Spatial association between PDGFRα (red) and Pecam-1 (white). Scale bars = 200 μm. (d–h) Analysis in PDGFRα reporter (tdTomato) mice. (d) PDGFRα-lineage cells visualized by tdTomato fluorescence enhanced with an anti-RFP antibody (red). Scale bar = 200 μm. (e) Integration of PDGFRα-lineage cells (red) with Pecam-1-positive vascular networks (white pseudocolor). Scale bar = 200 μm. (f) Higher magnification showing the perivascular localization of PDGFRα-lineage cells (red) around Pecam-1-positive vessels (white). Scale bar = 25 μm. (g, h) Magnified views of the boxed regions in (f) showing PDGFRα-lineage cells adjacent to (g, solid box) and encircled (h, dotted box) vessel walls. Scale bars = 25 μm. Quantification of panel (e) showed that 44.7% (51/114) of PDGFRα-lineage cells were in direct contact with vascular structures (within 5 μm of Pecam-1-positive vessels).

The cellular composition of mold-derived tissues from wild-type mice was examined using immunofluorescence. Staining revealed that PDGFRα-positive cells were dispersed within the tissue matrix (Fig 2a) and Pecam-1-positive vascular structures (Fig 2b). The visual assessment revealed a close spatial association between PDGFRα-positive cells and Pecam-1-positive vessels (Fig 2c), indicating a potential interaction. However, this was not formally quantified. PDGFRα reporter mice (tdTomato) were used to track the fate of PDGFRα-expressing cells. Immunostaining for red fluorescent protein (RFP) enhanced the visualization of PDGFRα-lineage cells (Fig 2d) compared with native fluorescence. The reporter cells were extensively integrated into the Pecam-1-positive vascular networks (Fig 2e). Quantitative analysis of a representative microscopic field revealed that 44.7% (51 of 114) of PDGFRα-lineage cells were in direct contact with Pecam-1-positive vascular structures, indicating their preferential perivascular localization. At higher resolution, PDGFRα-lineage cells were observed directly adjacent to vessel walls (Fig 2f and g) and encircling vessels in a perivascular manner (Fig 2f and h), indicating their role in supporting vascular formation or maturation. Examination of the tissue microenvironment in one PDGFRα reporter mouse (S1 Fig) suggested the presence of Type III collagen (S1a Fig), a component indicative of an immature connective tissue matrix often associated with tissue remodeling. In this animal, staining for ER-TR7, a marker of reticular fibroblasts and associated fibers [27], also revealed reticular fibers that appeared to provide structural support throughout the tissue (S1b Fig). Although PDGFRα-lineage cells were situated within this matrix, direct colocalization with ER-TR7 fibers was infrequent, indicating that these cells reside within this specific fibrous network but may not be the primary source of it.

### Flow cytometric analysis revealed distinct surface marker expression profiles

**Fig 3:**
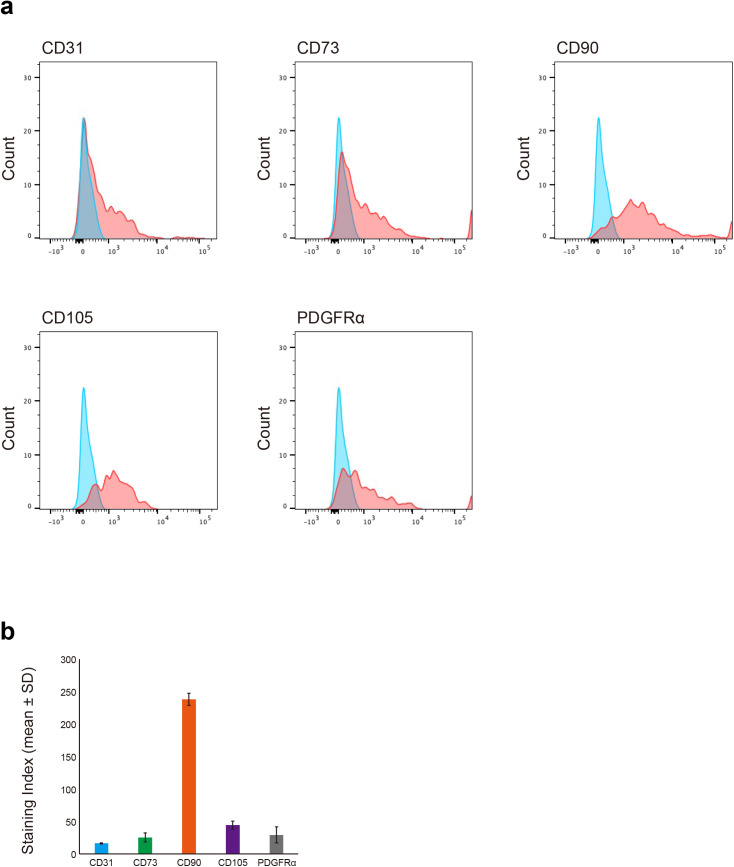
Flow cytometric characterization of surface markers in mold-derived cells. (a) Representative flow cytometry histograms showing the expression intensity of the endothelial marker Pecam-1 (CD31), MSC-associated markers (CD73, CD90, and CD105), and PDGFRα in dissociated cells from pooled mold-derived tissues. Blue-filled histograms represent isotype controls, and red-filled histograms represent specific antibody staining. (b) Quantitative analysis of marker expression presented as the SI (calculated as described in the Methods section). Bars represent the mean ± SD from three independent experiments, each analyzing pooled samples (n = 3 mice per pool).

We performed flow cytometry on dissociated cells to assess surface marker expression in cells within the mold-derived tissues. These freshly isolated cells represented a heterogeneous population, as detailed in the Methods section. Fig 3a shows the expression patterns of the endothelial marker Pecam-1 (CD31), the MSC-associated markers CD73, CD90, and CD105, and the in vivo MSC marker PDGFRα. Quantitative analysis using the staining index (SI), calculated from three independent experiments using pooled samples, revealed differential expression levels of these markers (Fig 3b). CD90 exhibited the highest SI (238.1 ± 9.3), indicating strong expression within the cell population. CD105, PDGFRα, and CD73 exhibited moderate SI (44.5 ± 6.1, 29.4 ± 12.3, and 25.6 ± 6.9, respectively). Conversely, the endothelial marker Pecam-1 exhibited a comparatively lower SI (16.3 ± 0.9). These results confirmed the presence of cells expressing PDGFRα and conventional MSC markers alongside endothelial cells within the iBTA-generated tissue.

### Cultured isolates from iBTA tissues exhibited MSC-like characteristics

**Fig 4:**
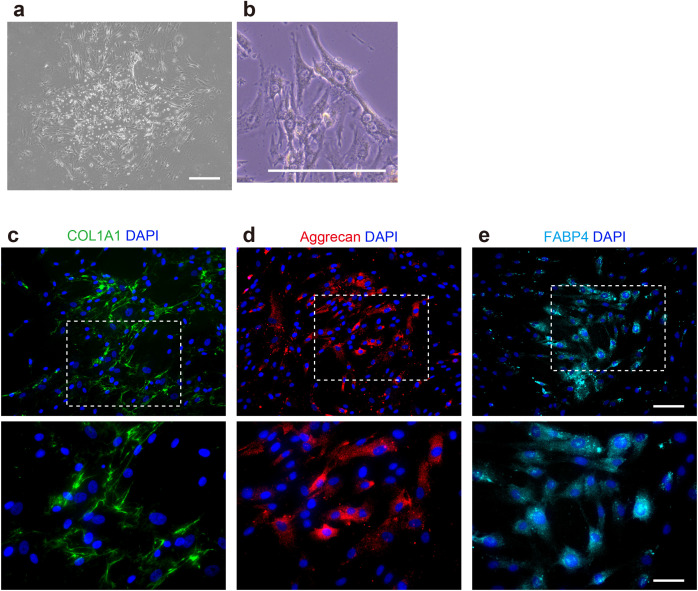
Morphological and differentiation characteristics of adherent cells isolated from iBTA tissues. All results shown are representative of n = 4 independent experiments demonstrating trilineage differentiation potential. (a, b) Representative phase-contrast images showing the typical spindle-shaped morphology of cultured cells at (a) low and (b) higher magnifications. Scale bars = 500 (a) and 200 μm (b). (c–e) Immunofluorescence detection of lineage-associated markers 14 days after induction. Upper panels show low-magnification views; lower panels show magnified views of the dashed boxed regions. (c) Osteogenic induction confirmed by COL1A1 expression (green). (d) Chondrogenic induction indicated by aggrecan expression (red). (e) Adipogenic induction indicated by FABP4 expression (cyan). The nuclei were counterstained with DAPI (blue). Scale bars = 100 μm (upper panels) and 50 μm (lower panels).

Adherent cell cultures were successfully established from enzymatically dissociated iBTA tissues and were expanded in vitro. These cells displayed a characteristic spindle-shaped morphology on both low- (Fig 4a) and high-magnification (Fig 4b) phase-contrast images, resembling typical cultured MSCs [28]. We assessed in vitro differentiation capacity using specific induction media (see Methods) for 14 days. Immunofluorescence staining was used to detect the expression of lineage markers. Osteogenic potential was suggested by the detection of the type I collagen protein COL1A1 [29,30] in induced cells (Fig 4c). The chondrogenic potential was indicated by aggrecan expression [31,32] (Fig 4d). The adipogenic potential was indicated by the presence of FABP4-expressing cells [33,34] (Fig 4e). These observations indicate that the cultured cell population derived from iBTA tissue retains multipotent differentiation capabilities consistent with MSCs based on immunofluorescence detection of these lineage-associated markers.

### Tissue formation and maturation occurred within iBTA molds in a porcine model

**Fig 5:**
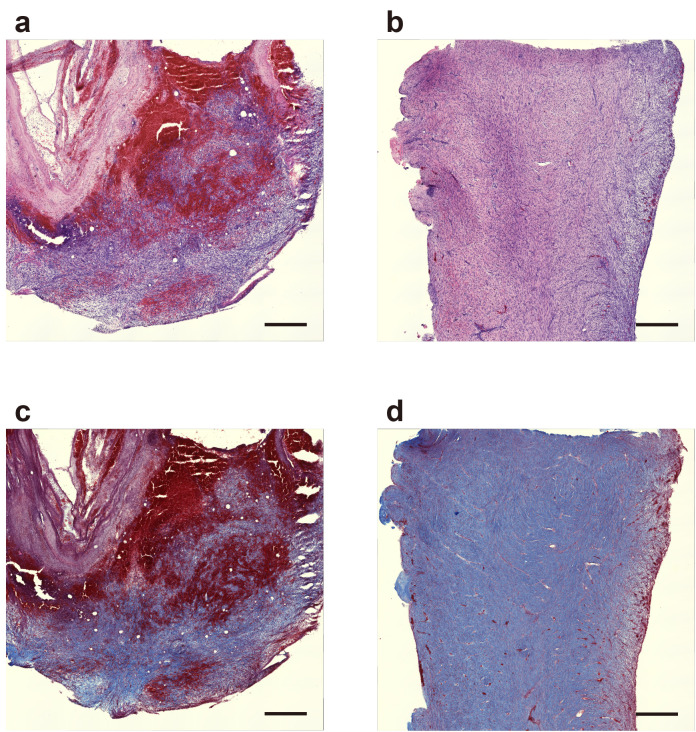
Histological analysis of tissue formation in molds implanted in domestic pigs. Representative histological images are illustrated.The images presented are from one representative animal chosen from a group of five animals that took part in the experiment. (a, b) HE staining showing cellular organization at (a) 1 and (b) 2 weeks after implantation. Progression from the initial cellular infiltration to a more organized tissue architecture was observed. Scale bar = 500 μm. (c, d) Masson’s trichrome staining revealed extracellular matrix development at (c) 1 and (d) 2 weeks after implantation. Blue staining indicated progressive collagen fiber deposition and organization. Scale bar = 500 μm.

We subcutaneously implanted molds in domestic pigs to assess the progression of tissue formation and maturation using iBTA in a large animal model relevant to clinical applications. Temporal changes were histologically analyzed. Hematoxylin and eosin (HE) staining revealed dynamic structural development within the implants. After 1 week of implantation, the tissue mass was characterized by densely packed cells and numerous vascular structures distributed throughout the matrix (Fig 5a). After 2 weeks, the tissue architecture showed increased maturity, with more organized cellular arrangements and evidence of stratification (Fig 5b). Masson’s trichrome staining revealed progressive extracellular matrix development. A marked increase in the density and organization of collagen fibers (stained blue) was observed from week 1 (Fig 5c) to week 2 (Fig 5d). The uniformly distributed and organized collagen network at 2 weeks indicated substantial matrix remodeling and tissue maturation within the mold-derived constructs.

## Discussion

Our investigation using the iBTA system revealed that endogenous PDGFRα-positive cells are key contributors to the de novo formation of vascularized tissue structures within subcutaneously implanted molds in mice. Furthermore, progressive tissue maturation was observed over 2 weeks in a clinically relevant porcine model, characterized by organized cellular arrangements and extensive matrix deposition. These findings indicate the potential of iBTA to generate autologous vascularized tissue constructs directly in vivo, thereby avoiding the common limitations of cell therapy approaches that require ex vivo culture and face immunocompatibility issues. Within the established tissue engineering framework—comprising scaffolds, growth factors, and stem cells—the iBTA mold functions as a scaffold that recruits and organizes endogenous cells in situ. Importantly, the primary objective of the present study was not to produce a clinically ready vascular graft but rather to elucidate the cellular mechanisms by which PDGFRα-positive cells contribute to autonomous vascular tissue formation within this scaffold-based platform. Functional evaluation of iBTA constructs as vascular grafts, including assessment of mechanical properties, patency, and thromboresistance, represents an important future direction that is beyond the scope of the current mechanistic investigation.

Endogenous PDGFRα-positive cells can organize into vascularized networks within the iBTA microenvironment in vivo. This contrasts with conventional tissue engineering strategies that typically rely on prefabricated scaffolds [35] and extensive ex vivo cell manipulation with defined cell populations [36]. The observed perivascular localization of PDGFRα-lineage cells strongly indicates their active participation in vascular development and stabilization within the iBTA construct, potentially through interactions with concurrently recruited endothelial progenitors, similar to the crosstalk between MSCs and endothelial colony-forming cells [37]. Moreover, preliminary observations from a single PDGFRα reporter mouse suggest that these lineage-traced cells may become integrated into a developing tissue matrix that includes Type III collagen (S1a Fig), potentially contributing to the structural integrity of the nascent tissue. These findings underscore the intrinsic vascular organizing potential of this specific MSC subset when mobilized in vivo.

Previous iBTA studies have focused on final-stage bulk tissue structures, which consisted primarily of collagen. The variability in tissue properties observed in earlier work may be explained by our current findings that PDGFRα-positive cells serve as key cellular contributors to tissue formation. Understanding this cellular basis provides opportunities for optimizing and standardizing the iBTA process.

This organizational capacity is consistent with those of other studies, which showed that in vitro models demonstrated the capacity of MSCs, when cocultured with endothelial cells, to generate stable microvascular networks that endure for extended periods within microfluidic systems [38]. Although direct comparisons are limited by the different environments, the persistence of vascular structures formed in vitro lends credence to the possibility that PDGFRα-positive cell-driven networks within the iBTA system possess inherent stability and potential for long-term durability in vivo.

Numerous preclinical studies on cell transplantation have supported the general concept that MSC populations contribute to vascular formation and repair. Transplanted MSCs have been shown to enhance angiogenesis and vascular remodeling in pelvic floor repair models [39]. Although these studies demonstrated the provascular potential of exogenously delivered MSCs, the findings of our study indicate that the iBTA system can leverage endogenously recruited PDGFRα-positive cells to achieve vascular organization in situ. This finding highlights the therapeutic potential inherent within resident MSC populations and aligns with the broader goal of harnessing MSCs for vascular regenerative strategies.

With recent advances in tissue engineering employing scaffolds, the versatility of MSCs in promoting vascular regeneration has been highlighted. Scaffolds incorporating MSC-derived factors enhance angiogenesis during wound healing [40], and biodegradable scaffolds seeded with MSCs enable long-term vascular structure formation [41]. Despite the effectiveness of such strategies, they require considerable ex vivo cell/factor preparation and scaffold fabrication. Conversely, our findings indicate that the iBTA method directly leverages the intrinsic vascular organizing capabilities of endogenous MSCs, particularly the PDGFRα-positive population, in vivo, potentially offering a more streamlined approach to vascular tissue engineering.

The ability of endogenous progenitor populations, such as PDGFRα-positive cells identified in this study, to contribute to vascular regeneration is consistent with the recognized roles of resident stem cells within vessel walls during tissue repair and remodeling [42]. These resident vascular stem cells can be activated by injury signals to participate in processes such as endothelial repair and neovessel formation [42]. This finding supports the principle of leveraging endogenous progenitor cells for tissue vascularization. The iBTA method facilitates the autonomous organization of recruited cells into vascularized structures without extensive ex vivo manipulation, representing a potentially significant advancement in regenerative strategies.

The analytical approaches for the murine and porcine models were tailored to the strengths of each system. In the mouse model, the availability of advanced genetic tools (e.g., PDGFRα-reporter mice) and a broad panel of specific antibodies enabled detailed cellular analyses focused on the role of PDGFRα-positive cells. In contrast, in the porcine model—where species-specific antibodies for immunofluorescence are more limited—we employed robust histological methods (H&E and Masson’s trichrome staining) to effectively assess overall tissue architecture and maturation.

In the porcine model, tissue development within iBTA molds showed clear progression over 2 weeks. At week 1, the initial phase involved substantial cellular infiltration and the presence of vascular structures within the matrix. By week 2, the tissue exhibited increased organization, stratification, and significant collagen deposition, indicating active tissue integration and maturation. This controlled tissue development in a porcine model strengthens its potential for clinical translation, particularly given that iBTA-generated autologous tissues have shown therapeutic efficacy in human applications, such as complex wound closure [16].

The clinical relevance of our findings is supported by parallels in regenerative medicine. The organization of PDGFRα-positive cells into vascularized structures via iBTA represents a significant advance, particularly considering the therapeutic efficacy of related cell populations, such as adipose-derived stromal vascular fraction cells [43,44]. The tissue organization observed in our model likely involves inherent MSC capabilities, such as differentiation toward perivascular mural cells expressing contractile proteins [45] and the production of extracellular matrix components essential for granulation tissue formation and vessel stabilization [46]. Furthermore, the known roles of PDGFRα signaling in connective tissue remodeling and injury responses [22,47] provide a mechanistic basis for how these specific cells orchestrate early tissue integration and vascular maturation within the iBTA environment, indicating the broad applicability of this approach in vascular regeneration. While our study demonstrates successful vascularization within iBTA constructs, we acknowledge that achieving complete endothelialization of the luminal surface remains a critical challenge for clinical translation. Future studies should focus on strategies to promote endothelial coverage of the inner surface to prevent thrombosis in vascular applications. An alternative experimental approach to demonstrate the importance of PDGFRα-positive cells would be cell depletion using Pdgfra-CreER/R26-DTA mice. However, recent evidence from Yang et al. [48] demonstrated that diphtheria toxin-mediated ablation of PDGFRα-positive cells in PdgfraCreER × DTA mice resulted in severe disruption of both dental pulp and periodontal ligament tissues, including collapse of the odontoblast layer and marked shrinkage of pulp core tissue, along with substantial loss of periodontal collagen fibers. These findings reflect the fact that PDGFRα occupies an apex position in the mesenchymal cell hierarchy [19], and its depletion simultaneously eliminates multiple downstream lineage-committed cell populations, thereby precluding the isolation of PDGFRα-specific contributions. For this reason, the present study adopted a single-arm lineage tracing approach focused on tracking PDGFRα-positive cells within the iBTA system, which enables direct observation of cell fate without confounding tissue-wide destruction. Complementary approaches, including single-cell RNA sequencing or spatial transcriptomics of PDGFRα-positive cells isolated from iBTA tissues, would provide further molecular insights into their functional roles and represent important future directions.

This study has some limitations. First, although the iBTA system effectively harnesses endogenous healing/regenerative mechanisms, individual response variability may present challenges in standardizing outcomes. Second, the sample size for certain qualitative immunofluorescence analyses in PDGFRα reporter mice was limited. While co-localization studies with Pecam-1 were conducted using tissues from three animals (n = 3), characterizations involving Type III collagen and ER-TR7 were performed using tissue from a single animal (n = 1). As such, observations related to these matrix components should be considered preliminary. Third, the assessment of trilineage differentiation potential was based on immunofluorescent detection of lineage-associated protein markers rather than traditional histological staining. Additionally, detailed histological analysis (H&E and Masson’s trichrome) was performed only in the porcine model. Furthermore, this histological analysis was qualitative. Future studies would benefit from quantitative assessments, including collagen density or vessel counts, to further substantiate the findings on tissue maturation. Fourth, the long-term stability and functional integration of engineered vascularized tissues were not investigated. Therefore, further investigation is needed to ensure the safety and efficacy of engineered vascularized tissues. Finally, the optimization of specific clinical applications and scale-up strategies requires additional development. Despite these limitations, the findings of this study establish a foundation for patient-specific vascular tissue engineering that circumvents the complications associated with traditional cell transplantation. The autonomous organization of PDGFRα-positive cells into vascularized tissue structures via iBTA represents a promising direction for treating conditions that require tissue regeneration.

## Methods

### Animals and ethical considerations

All animal experiments were approved by the Institutional Animal Care and Use Committees of the Keio University School of Medicine (Approval number: A2021-027, A2023-007, D2021-4) and Narita Animal Science Laboratory Co., Ltd. (Approval number: 22L-S070) and conducted in accordance with the ARRIVE guidelines and the relevant international standards (Animal Act 1986, EU Directive 2010/63/EU, and NIH Guide for the Care and Use of Laboratory Animals). Sample sizes for mouse experiments were determined based on previous experience with the model system and feasibility, ensuring sufficient tissue for planned analyses while adhering to the 3Rs principles of animal use (Replacement, Reduction, and Refinement). Particularly, immunofluorescence analyses involving PDGFRα reporter mice used tissues from confirmed genotype-positive animals.

### iBTA device preparation

Cylindrical porous stainless steel molds (Biotube Co., Tokyo, Japan) with silicone end caps and various dimensions were used (Fig 1b–d). The molds were sterilized by autoclaving and stored in 70% ethanol. Immediately before surgery, they were extensively rinsed with sterile physiological saline.

### Mouse model

C57BL/6J mice (Japan SLC, Shizuoka, Japan) and PDGFRα reporter mice were used in this study. For key analyses, experimental groups typically consisted of n = 3–5 animals; precise sample sizes for each experiment are detailed in the relevant Methods subsections and/or figure legends. The reporter line was generated by crossing *Pdgfr*α-*CreERT* mice (Jackson Laboratory, Bar Harbor, ME, USA; Stock No.: 018280) with *Rosa26-tdTomato* mice (Jackson Laboratory, Stock No.: 007914). Both male and female mice (8–10 weeks old at induction) were included in preliminary assessments, and no significant sex-dependent differences were observed in the outcomes measured. Recombination was induced by intraperitoneal injection of tamoxifen (100 mg/kg body weight in corn oil; Toronto Research Chemicals, Toronto, ON, Canada) administered on alternate days for a total of three doses between 13 and 15 weeks of age (Fig 1a). Animals were housed in polycarbonate cages (≤5 per cage) under controlled environmental conditions (12:12 h light/dark cycle, 23.5°C, and 55% humidity) with ad libitum access to water and a sterilized CE-2 diet (30-kGy irradiation; CLEA Japan, Tokyo, Japan). The cages were cleaned weekly.

The procedures were performed in 10-week-old mice. Animals were anesthetized with an intraperitoneal injection of a mixture of medetomidine (Domitor, Nippon Zenyaku Kogyo Co., Ltd.; 75 µg/mL), midazolam (Midazolam Sandoz, Sandoz K.K.; 400 µg/mL), and butorphanol (Vetorphale, Meiji Seika Pharma Co., Ltd.; 500 µg/mL). The adequacy of anesthesia was ensured by confirming muscle relaxation. The dorsal skin was shaved, and the surgical site was aseptically prepared using povidone-iodine and 70% ethanol. A 6.0 mm longitudinal incision was made slightly off the midline, and a subcutaneous pocket was created by blunt dissection. A single mold was inserted into the pocket. The skin incision was closed using 4-0 nylon sutures. The entire procedure, including suturing, was typically performed within 10 min.

To facilitate recovery from anesthesia, atipamezole hydrochloride (Antisedan, Nippon Zenyaku Kogyo Co., Ltd., Japan; 0.75 mg/kg) was administered intraperitoneally after surgery. Mice were monitored until key indicators of recovery—righting reflex, normal breathing, and purposeful movement—were observed before returning them to their home cages. Postoperative analgesia was not administered due to the minimally invasive nature and short duration of the surgical procedure. The surgical site was inspected daily for 7 days. Molds containing the formed tissue were harvested 6 weeks after implantation. All procedures were performed following the institutional guidelines for aseptic surgery.

### Porcine model

Female domestic pigs (n = 5, 3 months old; Ishige Farm, Chiba, Japan) were housed at the Narita Animal Science Laboratory (Chiba, Japan) under controlled conditions (20°C–28°C, 40%–60% humidity, 12 h light cycle). Elliptical cylindrical molds (64 × 25.6 mm) were implanted subcutaneously in the dorsum using standard sterile procedures. General anesthesia was induced with ketamine (2 mg/kg) and maintained with 2%–3% sevoflurane. Tissues were harvested at 1 and 2 weeks post-implantation for histological analysis.

### Immunofluorescence analysis

#### Tissue processing.

Molds containing tissues were harvested 6 weeks after implantation from n = 3 PDGFRα reporter mice (for analyses in Fig 2d–h; one animal used for S1 Fig) and n = 3 wild-type mice (for Fig 2a–c). Tissues were fixed in situ within the molds in 4% paraformaldehyde/phosphate-buffered saline (PBS) for 24 h, cryoprotected using a sucrose gradient of 10%–30% in PBS, removed from the molds, embedded in FSC 22 compound (Leica, Wetzlar, Germany), and sectioned at 10 µm using a CM3050S cryostat (Leica).

#### Immunostaining.

The sections were permeabilized with 0.2% Tween 20 in PBS for 15 min and blocked using 5% donkey serum in 1% BSA for 1 h. Primary antibody incubation was performed with goat anti-PDGFRα (AF1062, 1: 50; R&D Systems, Minneapolis, MN, USA), rabbit anti-RFP (ab62341, 1: 250; Abcam, Cambridge, UK), rat anti-Pecam-1 (sc-18916, 1: 50; Santa Cruz Biotechnology, Dallas, TX, USA), rat anti-ER-TR7 (ab51824, 1: 200; Abcam), and goat anti-collagen III (NBP1–26547, 1: 400; Novus Biologicals, Centennial, CO, USA) at 4°C for 16 h. The sections were washed and incubated at room temperature for 2 h with Alexa Fluor 488, 555, or 647-conjugated secondary antibodies (1: 500–1: 1000; Abcam or Thermo Fisher Scientific, Waltham, MA, USA) and DAPI (1 µg/mL; Nacalai Tesque, Kyoto, Japan).

#### Image acquisition and analysis.

Confocal microscopy was performed using a Zeiss LSM710 or LSM980 microscope equipped with an Airyscan detector. Images were acquired and processed using Zen software 3.8 (Zeiss) and Photoshop (Adobe). Qualitative analysis was performed, focusing on the presence, distribution, and colocalization patterns of the stained markers and visual assessment of the spatial relationship between PDGFRα-positive cells and Pecam-1-positive vascular structures.

### Flow cytometric analysis

Single-cell suspensions were prepared from mold-derived tissues and analyzed by flow cytometry. Briefly, tissues harvested from the molds were pooled (n = 3 mice per pool) for each independent experiment and were mechanically disrupted and enzymatically digested with Type IV collagenase (10,000 U/mL; Gibco, Thermo Fisher Scientific, Waltham, MA, USA). After red blood cell lysis with Red Blood Cell Lysis Buffer (Roche, 11814389001, Mannheim, Germany) and washing, the cells were stained with APC-conjugated monoclonal antibodies for 30 min at 4°C. An antibody panel was used to assess the expression profile of MSC markers (CD73, CD90, and CD105), the in vivo MSC marker PDGFRα (CD140a), and the endothelial marker CD31 within the recruited cell population, including anti-CD31 (clone 390, #102410), anti-CD73 (clone TY/11.8, #127209), anti-CD90.2 (clone 30-H12, #105311), anti-CD105 (clone MJ7/18, #120413), and anti-CD140a/PDGFRα (clone APA5, #135907) (all from BioLegend). APC Mouse IgG2a, κ (clone X40, #567155; BD Pharmingen) was used as the isotype control. Notably, the analyzed cells were derived directly from dissociated heterogeneous tissue, which, as expected in an in vivo implant model, contained various cell types, including hematopoietic cells (CD45-positive). While this study focused on the expression of positive MSC-associated markers (CD73, CD90, CD105) and PDGFRα within this mixed population, full characterization to exclude hematopoietic cells—according to the International Society for Cellular Therapy criteria for cultured MSCs—was not the primary objective of this particular flow cytometric panel. Data were acquired using FACS Aria II (BD Biosciences). The SI was calculated to quantify the relative expression levels as follows: SI = (Median_positive − Median_isotype) / (2 × SD_isotype). Representative histograms, SI quantification (mean ± SD of three independent experiments using pooled samples) are shown in Fig 3a and 3b, respectively. The gating strategy is shown in S2 Fig. The antibody panel was selected based on International Society for Cellular Therapy (ISCT) criteria for MSC characterization, including positive markers (CD73, CD90, CD105) and the in vivo MSC marker PDGFRα.

### Differentiation potential analysis

Adherent cells obtained after enzymatic dissociation (using collagenase) were expanded in vitro using a mesenchymal stem cell growth medium (PT-3001, Lonza, Basel, Switzerland) to evaluate the multipotency of the cells recruited into the molds. The cultured isolates exhibited a typical spindle-shaped morphology (Fig 4a). Trilineage differentiation potential—osteogenic, chondrogenic, and adipogenic—was assessed and confirmed for each lineage in n = 4 independent experiments. Trilineage differentiation was induced by culturing the cells for 2 weeks in commercially available media from Lonza, including osteogenic medium (PT-3002), chondrogenic medium (PT-3003 supplemented with 10 ng/mL TGF-β3, PT-4124), and adipogenic medium (PT-3004). Differentiation outcomes were assessed by immunofluorescence detection of lineage-associated markers, such as COL1A1 (72026S, Cell Signaling Technology, Danvers, MA, USA) for osteogenesis, aggrecan (13880-1-AP, Proteintech Group) for chondrogenesis, and FABP4 (12802-1-AP, Proteintech Group) for adipogenesis. The stained sections were counterstained with a DAPI-containing mounting medium (ab104139; Abcam) and analyzed using a BZ-X800 fluorescence microscope (Keyence, Osaka, Japan).

### Histological analysis of porcine tissues

Porcine tissue samples harvested 1 and 2 weeks after implantation were fixed in 4% paraformaldehyde in PBS for 24 h at 4°C for morphological preservation. The samples were then processed using standard histological procedures, embedded in paraffin, and sectioned serially at a 10 μm thickness. The sections were stained with HE to evaluate the general tissue structure and cellular organization. Adjacent sections were stained with Masson’s trichrome staining kit (Muto Pure Chemicals Co., Ltd., Tokyo, Japan), following the manufacturer’s instructions, to visualize and assess the distribution and density of collagen fibers (connective tissue matrix). The stained sections were examined under a microscope (Keyence, Osaka, Japan), and images were captured.

## Supporting information

S1 Fig Preliminary characterization of extracellular matrix components in iBTA-generated tissue from a PDGFRα reporter mouse (n = 1). (a) Representative immunofluorescence image showing tdTomato-positive PDGFRα-lineage cells (red) and Type III collagen (green). (b) Representative immunofluorescence image showing tdTomato-positive PDGFRα-lineage cells (red) and ER-TR7-positive reticular fibers (white pseudocolor). Nuclei were counterstained with DAPI (blue). Both images are from tissue harvested 6 weeks after mold implantation from a single animal. Scale bars = 200 μm.
(TIF)

S2 Fig Representative flow cytometry gating strategy for mold-derived cells. (a) The main cell population (P1) was gated from total events based on forward scatter area (FSC-A) and side scatter area (SSC-A) to exclude debris. (b, c) Single cells were sequentially gated from the P1 population to exclude doublets: first using side scatter height (SSC-H) versus width (SSC-W) (b), followed by forward scatter height (FSC-H) versus width (FSC-W) (c). The final gated single-cell population was used for the marker expression analyses shown in Fig 3.
(TIF)

S1 Data Quantitative data for flow cytometric staining index analysis. (XLSX)
