# Supplementary figures and images for: PDGFRα-positive mesenchymal stem/stromal cells contribute to autonomous vascular formation through in-body tissue architecture
Source: PLoS One. 2026 Apr 16;21(4):e0347197. doi: 10.1371/journal.pone.0347197 (PMC13086343; doi:10.1371/journal.pone.0347197)

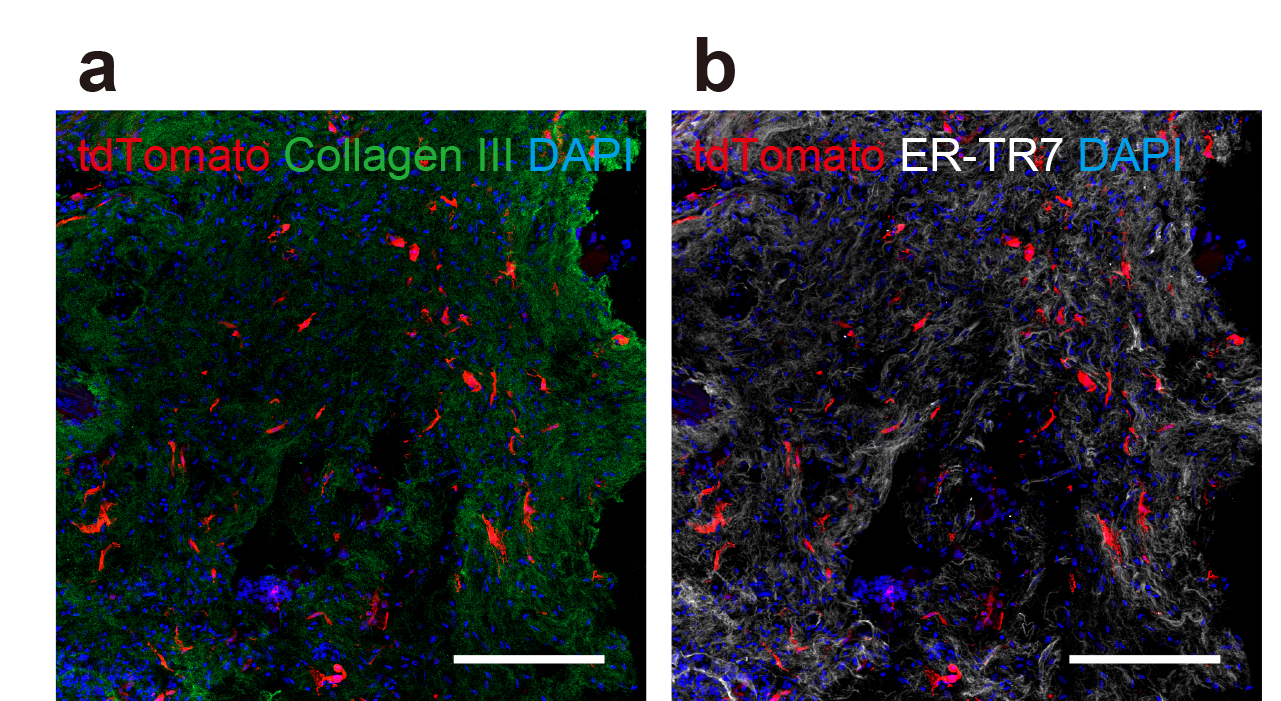

Supplement: S1 Fig — (a) Representative immunofluorescence image showing tdTomato-positive PDGFRα-lineage cells (red) and Type III collagen (green). (b) Representative immunofluorescence image showing tdTomato-positive PDGFRα-lineage cells (red) and ER-TR7-positive reticular fibers (white pseudocolor). Nuclei were counterstained with DAPI (blue). Both images are from tissue harvested 6 weeks after mold implantation from a single animal. Scale bars = 200 μm. (TIF) [file pone.0347197.s001.tif]

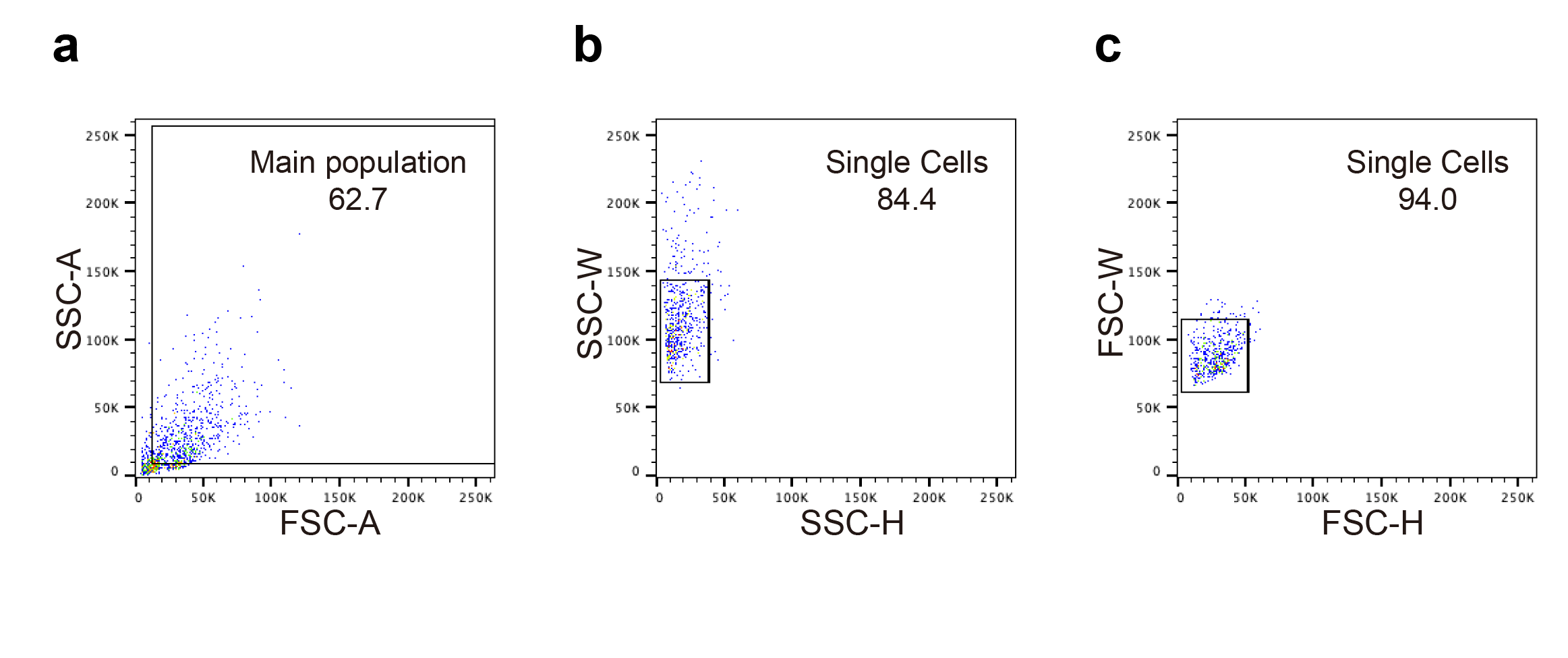

Supplement: S2 Fig — (a) The main cell population (P1) was gated from total events based on forward scatter area (FSC-A) and side scatter area (SSC-A) to exclude debris. (b, c) Single cells were sequentially gated from the P1 population to exclude doublets: first using side scatter height (SSC-H) versus width (SSC-W) (b), followed by forward scatter height (FSC-H) versus width (FSC-W) (c). The final gated single-cell population was used for the marker expression analyses shown in Fig 3. (TIF) [file pone.0347197.s002.tif]
